# Genomic landscape of malignant phyllodes tumors reveals multiple targetable opportunities

**DOI:** 10.1093/oncolo/oyae218

**Published:** 2024-08-27

**Authors:** Laura H Rosenberger, Richard F Riedel, Emilia J Diego, Amanda L Nash, Juneko E Grilley-Olson, Natalie A Danziger, Ethan S Sokol, Jeffrey S Ross, Sarah L Sammons

**Affiliations:** Department of Surgery, Duke University Medical Center, Durham, NC 27710, United States; Duke Cancer Institute, Duke University, Durham, NC 27710, United States; Duke Cancer Institute, Duke University, Durham, NC 27710, United States; Department of Medicine, Duke University Medical Center, Durham, NC 27710, United States; Department of Surgery, University of Pittsburgh Medical Center, Pittsburgh, PA 15213, United States; Department of Surgery, Duke University Medical Center, Durham, NC 27710, United States; Duke Cancer Institute, Duke University, Durham, NC 27710, United States; Department of Medicine, Duke University Medical Center, Durham, NC 27710, United States; Foundation Medicine, Inc., Cambridge, MA 02141, United States; Foundation Medicine, Inc., Cambridge, MA 02141, United States; Foundation Medicine, Inc., Cambridge, MA 02141, United States; Department of Pathology, Urology, and Oncology, Upstate Medical University, Syracuse, NY 13210, United States; Medical Oncology, Dana-Farber Cancer Institute, Harvard Medical School, Boston, MA 02215, United States; Breast Oncology Program, Dana-Farber Brigham Cancer Center, Boston, MA 02215, United States; Harvard Medical School, Boston, MA 02115, United States

**Keywords:** phyllodes tumor, malignant phyllodes, genomic sequencing, genomic mutation, targeted therapy, *TERT* promoter

## Abstract

**Background:**

Malignant phyllodes tumors (MPT) are rare fibroepithelial breast cancers with no known effective systemic therapy; metastatic progression portends a dismal prognosis. We sought to describe the genomic landscape of MPTs through genomic profiling and immunotherapeutic biomarker analysis.

**Materials and methods:**

Cases of sequenced MPT were identified from a Clinical Laboratory Improvement Amendments-certified, College of American Pathologists-accredited laboratory (Foundation Medicine). All cases underwent genomic profiling using adaptor ligation-based, next-generation sequencing assay of 324 genes. Tumor agnostic immunotherapy biomarkers, microsatellite instability, tumor mutational burden (TMB), and programmed death-ligand 1 (PD-L1) expression were evaluated. Fisher’s Exact Tests and analysis of variance were used to test for differences between groups and for continuous variables as appropriate.

**Results:**

Of 135 MPT cases identified; 94 (69.6%) were localized/locally recurrent and 41 (30.4%) were metastatic. Median age was 54 years (range 14-86). The median TMB was 2.5 mut/Mb and 3 were TMB-high (≥10 mut/Mb). 21.4% were PD-L1+ via Dako 22C3 assay (CPS ≥1). Most commonly altered genes included *TERT-*promoter (69.7%)*, CDKN2A* (45.9%), *TP53* (37.8%), *NF1* (35.6%), *CDKN2B* (33.3%), *MED12* (28.9%), *MTAP* (27.7%), *KMT2D* (22.2%), *PIK3CA* (20.0%), *PTEN* (18.5%), and *RB1* (18.5%). Several tumors harboring genomic alterations with US Food and Drug Administration-approved indications in other tumor types were found including *NF1*, *PIK3CA, EGFR* Exon 19/20 insertions, and *BRAF V600E* mutations.

**Conclusions:**

In the largest genomic evaluation of MPT to date, multiple clinically actionable mutations were found. Routine sequencing of metastatic MPT may provide additional information to guide treatment decisions and clinical trial enrollment.

## Teaser

This genomic study of malignant phyllodes tumors identified multiple clinically actionable mutations and biomarkers for immunotherapy response. Many of these genomic alterations have FDA-approved indications in other tumor types, offering potential targeted therapy options.

**Implications for practice**

This genomic study of malignant phyllodes tumors (MPT) identified multiple clinically actionable mutations and biomarkers for immunotherapy response. Many of these genomic alterations have US Food and Drug Administration-approved indications in other tumor types, offering potential targeted therapy options. Routine sequencing is not currently performed and national guidelines are devoid of recommendations for systemic therapy, which is not often offered in the non-metastatic setting. These findings suggest that investigating immunotherapeutic strategies could be beneficial for women with MPT, especially when metastatic, and provide information supporting routine sequencing of metastatic MPT to guide treatment decisions and clinical trial enrollment.

## Introduction

Phyllodes tumors (PTs) are rare fibroepithelial breast neoplasms characterized by epithelial and stromal histologic components,^1-3^ and are subclassified by the World Health Organization into 3 categories; (1) benign, (2) borderline, and (3) malignant. While benign PTs are clinically indolent, malignant phyllodes tumors (MPTs) have an aggressive biological behavior that disproportionately affects young women (median age of 45)^4^ and notably high local recurrence (LR) rates and metastatic potential. In large series, LR rates are reported as 8.0%-10.9% for benign, 13.0%-14.4% for borderline, and 18.0%-20.6% for MPT.^5-8^ These LR rates are markedly higher than the typical cancer of the breast, invasive adenocarcinoma, owing to the multitude of highly effective systemic therapy options for breast adenocarcinoma, frequently given in the adjuvant, non-metastatic setting. Distant metastases rarely occur in benign and borderline PTs (0.1% and 1.6%, respectively); however, they are a frequent occurrence in MPTs at 16.7%-22%.^1,8^

Despite known high LR and metastatic rates, treatment is almost exclusively surgical, as there are no known effective chemotherapy or targeted systemic therapy options.^9^ Metastatic progression, which occurs frequently (20%), portends a dismal prognosis; median survival is just 7-15 months.^10,11^ Current national guidelines for MPT are devoid of any systemic therapy recommendations, and systemic therapy is not offered in the non-metastatic setting. Management of metastatic MPTs follows principles of soft tissue sarcoma (STS), which do not recommend biomarker testing in the non-metastatic setting, precluding the ability to either predict or prevent distant relapse. The STS guidelines generally recommend next-generation sequencing on an individual basis only for patients who may qualify for clinical trials or are refractory to systemic therapies.^12^ Notably, STS trials that form the basis of the national guidelines typically do not include MPT as a sarcoma subtype.

In an effort to identify therapeutic targets in MPTs, research with *limited* series has discovered potentially actionable mutations in the RTK/RAS/RAF (*NF1, EGFR, BRAF, NRAS)* and PI3K/AKT/mTOR (*PIK3CA, PTEN, STK11*) pathways^13-25^; and while there are US Food and Drug Administration (FDA)-approved therapies available in other tumor types, these have never been tested in MPTs. Furthermore, oncologists rarely sequence MPTs until after progression of metastatic disease on first-line chemotherapy, due to (1) a lack of data regarding targeted therapy efficacy, (2) uncertainty regarding insurance authorization with potential for direct patient financial toxicity, and (3) a lack of supporting guidelines.

In the largest genomic profiling effort of MPTs to date, we sought to describe the genomic landscape of MPTs through genomic profiling and immunotherapeutic biomarker analysis in a US-based population.

## Materials and methods

Approval for this study, including a waiver of informed consent and Health Insurance Portability and Accountability Act waiver of authorization, was obtained from the Western Institutional Review Board (IRB) (Protocol #20152817), in addition to IRB approval at Duke University (IRB# Pro00108925). All cases of sequenced MPT were identified and analyzed from a database of real-world patients, tested as part of routine clinical care and sequenced in a Clinical Laboratory Improvement Amendments-certified, College of American Pathologists-accredited laboratory (Foundation Medicine, Cambridge, MA). This study included patients of any age, sex, race, or ethnicity and as a cohort study including all possible cases available in the database did not have any therapeutic intervention, randomization, blinding, or power analysis. The pathologic diagnosis of MPT was confirmed based on a hematoxylin and eosin (H&E) stained slide, with confirmation of ≥20% tumor nuclei per case prior to sequencing to ensure adequate sensitivity for alteration detection. Genomic profiling was performed by DNA extraction from formalin-fixed paraffin-embedded (FFPE) samples using a hybrid capture, adaptor ligation-based next generation sequencing assay as previously described.^26,27^ Detection of all classes of genomic alterations were identified, including substitutions, insertion and deletion alterations (indels), copy number alterations (CNAs), and select gene rearrangements in 324 genes. Only known or likely pathogenic alterations were included for analysis in this study. Variants of unknown significance were excluded when evaluating alteration frequencies and targetability of alterations. In addition, genomic signatures, including microsatellite instability (MSI) and tumor mutational burden (TMB), were evaluated as previously described.^28,29^ Programmed Cell Death Ligand 1 (PD-L1) expression by immunohistochemistry (IHC) was measured by the DAKO 22C3 assay in some cases (positive indicating tumor cell positive score ≥1). Tumor proportion score equals the number of PD-L1 staining tumor cells divided by the total number of viable tumor cells, multiplied by 100. PD-L1 expression was most often assessed by the Ventana SP142 assay (positive indicating ≥1% tumor immune cells express PD-L1).

Cases were categorized as localized/locally recurrent (“breast”) or metastatic (eg, “lung,” “bone,” “liver,” etc.), based on specimen site, and excluded from specific location analyses when indeterminant (eg, “soft tissue,” “chest wall”). Patient characteristics and results of genomic profiling of MPT were summarized by either N (%) or median (range). Factors from MPT were compared to all cases of breast carcinoma in the clinical database. Fisher’s Exact Tests were used to test for differences between groups and analysis of variance was used to test for differences for continuous variables.

## Results

### Demographics of MPT cohort

We identified 135 consecutive cases of MPT; 94 (69.6%) were classified as being presumed localized/locally recurrent (L/LR) and 41 (30.4%) were definitively metastatic. Sites presumed to be localized included: 51.1% breast (*N* = 69), followed by chest wall (10), soft tissue (9), skin (3), or lymph node (3), while metastatic sites were 73% lung (*N* = 30), followed by bone (4), liver (2), peritoneum (1), pleura (1), other intra-abdominal sites (3). All patients were female with a median age of 54 years (range 14-86). Predominant ancestry-informative markers identified this population as 60.8% (*N* = 82) European descent (EUR), 13.6% (*N* = 18) Admixed American (AMR, native North or South American), 11.2% (*N* = 15) African (AFR), 9.6% (*N* = 13) East Asian (EAS), and 4.8% (*N* = 6) South Asian (SAS).

### Genomic alterations and immune biomarker frequencies in MPT cohort

We identified a total of 646 genomic alterations, identified in 90 genes within the 135 cases of MPT sequenced. The median age of women with metastatic disease was significantly older than those with L/LR disease (49.5 vs. 60 years, *P* < 0.0001). The median TMB across samples was 2.5 mut/Mb (range 0-12 mut/Mb), which was similar between L/LR and metastatic cases (*P* = .32). Only 3 cases (1.45%) were TMB-high (≥10mut/Mb). 36.8% of samples evaluated in the whole cohort (*N* = 19) were PD-L1 positive via Ventana SP142 assay (≥1% immune cells) and 21.4% (*N* = 14 evaluated) were PD-L1 positive via Dako 22C3 assay (TPS ≥1). All evaluable cases (*N* = 132) were microsatellite stable (Table 1). There were no differences in median TMB, TMB-High, or PD-L1 positivity between definitively L/LR (specimens from the breast) versus metastatic specimens.

**Table 1.** Malignant phyllodes tumor patient demographics, mutational burden, and PD-L1 status, by specimen location.

	Local/locally recurrent *N* = 69^*^	Metastatic *N* = 44	*P*-value
Median age, years	49.5	60.0	**<.0001**
Ancestry			
AMR ancestry	13.9%	15.0%	1
EUR ancestry	58.5%	65.0%	.54
EAS ancestry	9.2%	12.5%	.74
SAS ancestry	4.6%	5.0%	1
AFR ancestry	13.9%	2.5%	.08
Median TMB	2.50	2.50	.32
TMB ≥ 10	1.5%	0.0%	1
MSI-H	0.0%	0.0%	1
# With PDL1 22C3 CPS	***N* = 4**	***N* = 8**	
PD-L1 22C3 TPS: negative	75.0%	87.5%	1
PD-L1 22C3 TPS: low positive	0.0%	12.5%	1
- PD-L1 22C3 TPS: high positive	25.0%	0.0%	.33
# With PDL1 SP142 IC	***N* = 10**	***N* = 7**	
PD-L1 SP142 IC: negative	70.0%	57.1%	.64
PD-L1 SP142 IC: positive	30.0%	42.9%	.64

^*^Includes only definitively local/locally recurrent cases (specimen site designated as “breast”).

Abbreviations: AMR, admixed American; AFR, African; EAS, East Asian; EUR, European; MSI, microsatellite instability; NS, non-significant; PD-L1, programmed cell death ligand 1; SAS, South Asian; TMB, tumor mutational burden. Low positive (1%-49%), high positive (50%+), .

**Figure 1.**
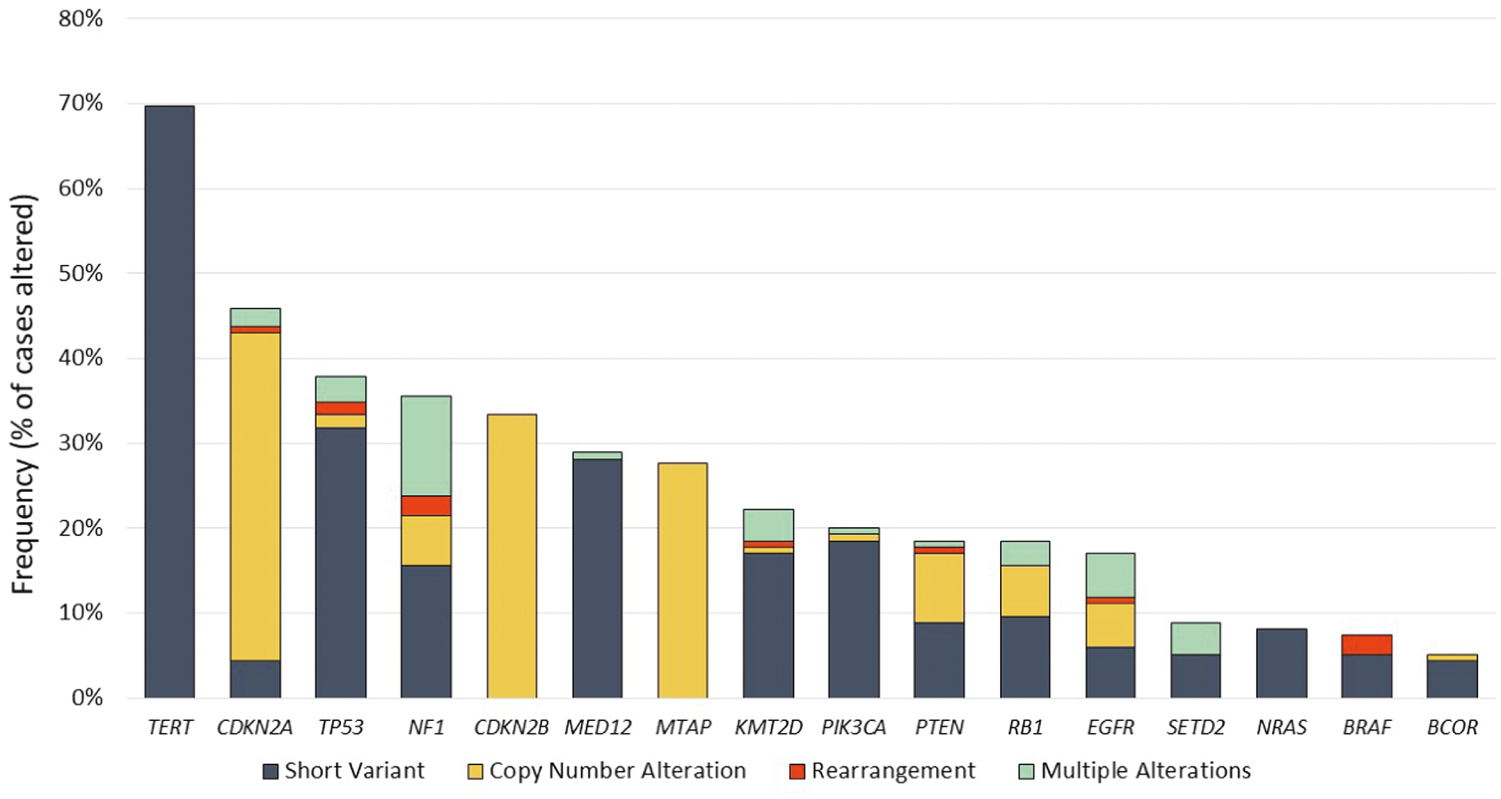
Longtail plot of genomic alterations identified in 135 cases of malignant phyllodes tumors with frequency identified in greater than 5% of samples.

**Figure 2.**
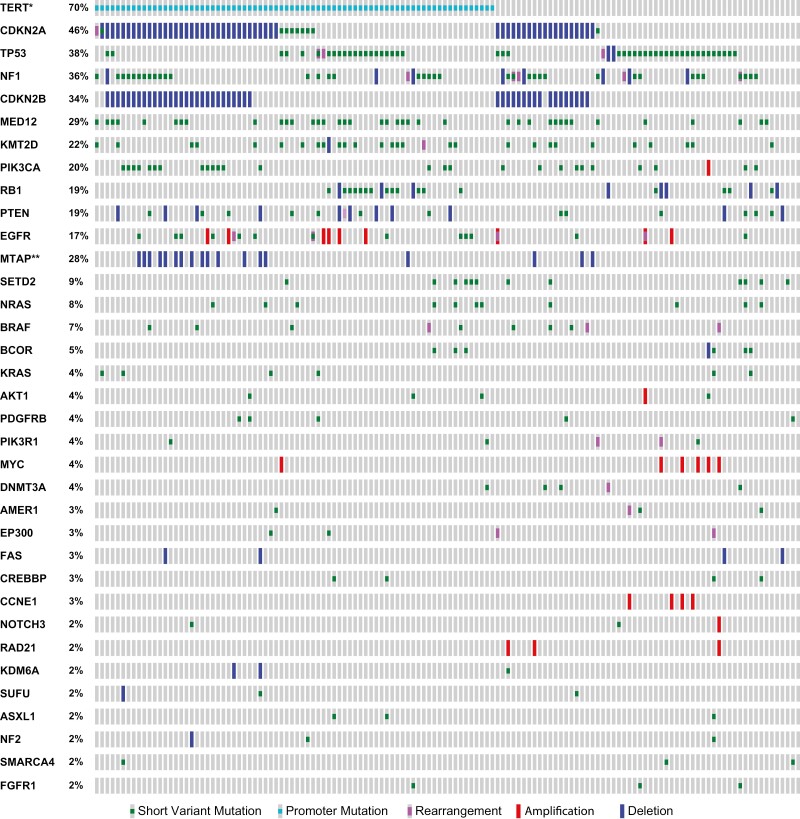
Oncoprint of genomic alterations identified in 135 cases of malignant phyllodes tumors with frequency identified in greater than 2% of samples.

The most commonly altered genes in this cohort, including all genes with a mutational frequency of ≥5% were *TERT-*promoter (69.7%)*, CDKN2A* (45.9%), *TP53* (37.8%), *NF1* (35.6%), *CDKN2B* (33.3%), *MED12* (28.9%), *MTAP* (27.7%), *KMT2D* (22.2%), *PIK3CA* (20.0%), *PTEN* (18.5%), *RB1* (18.5%), *EGFR* (17.0%), *SETD2* (8.9%), *NRAS* (8.1%), *BRAF* (7.4%), *BCOR* (5.2%); notably distinct in frequency from the breast carcinoma cohort (Figures 1 and 2, Table 2). When evaluating the mutational frequencies between alterations in definitively L/LR (specimen site designated as “breast”) versus metastatic specimens, there were significantly more frequent mutations identified in *NRAS* (4.4% vs 18.2%, *P* = .022) and *TERT* (63.2% vs 83.8%, *P* = .037) in the metastatic tumors.

**Table 2.** Frequency of genomic alterations identified in 135 cases of malignant phyllodes tumors.

Gene	Mutations in phyllodes cohort (*N* = 135)	Mutations in phyllodes cohort (%)	Mutations in breast cohort (*N* = 44 798)	Mutations in breast cohort (%)	*P*-value
*TERT* ^*^	76	69.7	467	1.1	<.001
*CDKN2A*	62	45.9	3137	7.0	<.001
*TP53*	51	37.8	23315	52.0	<.001
*NF1*	48	35.6	2954	6.6	<.001
*CDKN2B*	45	33.3	2258	5.0	<.001
*MED12*	39	28.9	89	0.2	<.001
*MTAP* ^**^	18	27.7	1021	3.7	<.001
*KMT2D*	30	22.2	943	2.1	<.001
*PIK3CA*	27	20.0	16062	35.9	<.001
*PTEN*	25	18.5	5735	12.8	.053
*RB1*	25	18.5	3479	7.8	<.001
*EGFR*	23	17.0	1117	2.5	<.001
*SETD2*	12	8.9	451	1.0	<.001
*NRAS*	11	8.1	272	0.6	<.001
*BRAF*	10	7.4	667	1.5	<.001
*BCOR*	7	5.2	232	0.5	<.001

^*^ *N* = 109 in phyllodes cohort, *N* = 42 382 in breast carcinoma cohort.

^**^ *N* = 65 in phyllodes cohort.

Table excludes genes with fewer than 5% of cases in phyllodes cohort.

Alterations in genes affecting cell cycle regulation (eg, *CDKN2A*/B and *TP53)* were mutually exclusive from one another (*P* < .001). *CDKN2A/B* alterations frequently co-occurred with MTAP (*P* < .001), as did *NRAS* with *SETD2* (*P* < .001). No *ALK*, *RET*, *ROS1*, or *NTRK* fusions were detected in any of the MPT samples, but an *FGFR3* fusion was found in one case (*FGFR3-TACC3* fusion).

### Prevalence of potentially therapeutically actionable alterations in MPT cohort

Several tumors harboring genomic alterations with FDA-approved indications in other tumor types were found including pathogenic *PIK3CA, EGFR* Exon 19/20 insertions, *KRAS* G12C (*N* = 1), *FGFR* fusions (*N* = 1), *BRAF V600E* mutations (*N* = 4), and *BRCA2* (*N* = 1) (Table 3). Hotspot *PIK3CA* mutations were found in E542 and E545 in the helical domain (exon 9, *N* = 7) and H1047 in the kinase domain (exon 20, *N* = 13). Additional alterations observed in *PIK3CA* included: C420R (*N* = 2), E110del (*N* = 1), H450_I459del (*N* = 1), L453_I459del (*N* = 1), and Q546L (*N* = 1). In addition to the exon 19/20 insertions seen in 4 cases, V774M alterations were seen in 2 samples, and the following *EGFR* short variant alterations were also found in one case each: G63R, I740V, R324L, A289V, Q432K, S229C, E114K, L63R, G719A, and H773R. The patient with a *BRCA2* alteration had frameshift at the S2509 residue (S2509fs*15 alteration).

**Table 3.** FDA-approved therapies by biomarker/genomic alteration identified in malignant phyllodes tumor cohort.

Tumor types	Biomarker/alteration	FDA-approved therapy
Solid tumor	TMB ≥10 mut/mb	Keytruda (pembrolizumab)
Breast cancer	*PIK3CA; various* C420R, E542K, E545A, E545D, E545G, E545K, Q546E, Q546R, H1047L, H1047R, H1047Y	Piqray (alpelisib)
Breast cancer	*ERBB2* (HER2) amplification	Herceptin (traztuzumab), Kadcyla (ado-trastuzumab-emtansine), or Perjeta (pertuzumab)
Breast, prostate and ovarian cancer	*BRCA 1/2 alterations*	Lynparza (olaparib) or Rubraca (rucaparib), or Zejula (niraparib)
Cholangiocarcinoma	*FGFR2* fusions	Pemazyre (pemigatinib) or Truseltiq (infigratinib or futibatinib)
Melanoma and non-small cell lung cancer	*BRAF* V600E or V600K	*BRAF* inibitors, Mekinist (trametinib), or BRAF/MEK inhibitor
Neurofibroma	*NF1* (oncogenic mutations)	Koselugo (selumetinib)
Non-small cell lung cancer	*EGFR* exon 19 del & *EGFR* exon 21 L858R alterations	*EGFR* tyrosine kinase inhibitors (TKI)
Non-small cell lung cancer	*EGFR* exon 20 T790M alterations	Tagrisso (osimertinib)
Non-small cell lung cancer	*BRAF* V600E	Tafinlar (dabrafenib) in combo w/ Mekinist (trametinib)
Non-small cell lung cancer	*KRAS* G12C	Lumakras (sotorasib), Krazati (adagrasib)

Abbreviations: FDA, US Food and Drug Administration; TMB, tumor mutational burden.

## Discussion

Over the last decade, there has been investigation into the genomic landscape of the broad spectrum of benign, borderline, and malignant phyllodes tumors in attempts to distinguish diagnosis and pathogenicity. In 2015, an early landmark study performed whole-exome sequencing of 22 PT (4 MPT) and 100 fibroadenomas (FA), with additional targeted deep sequencing for validation of prevalence, including 79 PTs.^13^ This identified *MED12* and *RARA* mutations were frequently observed (60%-80%) in both fibroepithelial tumors (PT and FA), emphasizing the importance of these mutations in fibroepithelial tumorigenesis.^13^ Additionally, PT also exhibited mutations in *FLNA, KMT2D,* and *SETD2,* suggesting their role in driving PT development and potential neoplastic transformation.^13^ Notably, higher-grade PT (borderline and malignant PTs) harbored additional mutations in cancer-associated genes, including *EGFR, ERBB4, NF1, PIK3CA, RB1,* and *TP53*.

These initial findings from 2015 have been validated in a plethora of subsequent small institutional series. Increasingly, studies have validated similar gene mutations in PT including *TERT, TP53, RB1, ERBB4, IGF1R, BRCA2, BCOR, MAP3K1, ZNF703,* and *TSC1*, as well as genes in the RTK/RAS/RAF pathway (*NF1, NRAS, BRAF, EGFR, FGFR1, FGFR3, AXL, KIT, PDGFRA*), and PI3K/AKT/mTOR pathway (*PIK3CA, PTEN, STK11, AKT1, TSC2*).^13-25^ While some of these are identified much more frequently in borderline/malignant PTs (*EGFR, ERBB4, NF1, PIK3CA, RB1, TERT, TP53*), others have been identified nearly exclusively in MPTs (*NF1, RB1, TP53*).^16^ Many of these genomic alterations, such as *BRAF, EGFR, PIK3CA,* and *NF1,* have a FDA-approved therapy in other tumor types. The abundance of multiple potentially targetable genomic alterations in MPTs provides a foundation for clinical trial design and further investigation of therapeutic targets. A major limitation with these prior genomic studies, however, is the limited number of MPTs analyzed, ranging from just 5 to at most, 24 MPTs included.

Herein, we present the largest series of genomic profiling of MPTs to date, including 135 consecutive cases of MPT, of which the majority harbored a potentially targetable alteration. For the first time, we describe the incidence of biomarkers for immunotherapy efficacy in solid tumors, in MPTs. Tumor agnostic biomarkers of immune checkpoint inhibitor (ICI) efficacy in solid tumors are high TMB and MSI. MPTs have relatively low median TMB and all were microsatellite stable, indicating ICIs alone may not be effective strategies. PD-L1 positivity in advanced triple-negative breast cancer (TNBC) is a marker of ICI efficacy when added to chemotherapy in the first-line setting. In our study, nearly a third of tumors were PD-L1 positive using criteria from pivotal phase III TNBC studies for immunotherapy efficacy.^30,31^ Prospective studies in PD-L1 positive MPT are needed to understand if this biomarker is relevant.

A third of cases harbored *NF1* gene alterations, which have been associated with clinical response to the MEK inhibitor selumetinib in those with *NF1*-related inoperable neurofibromas.^32^ Additional gene alterations in the RTK/RAS/RAF pathway, including *NRAS* and *BRAF,* were frequently identified (5%-15%) and have multiple FDA-approved therapies in other tumor types, as listed in Table 3. Activating *PIK3CA* mutations were found in 20% of tumors, which has significant implications in advanced breast adenocarcinoma. *PIK3CA*-mutated, hormone sensitive patients with HER2-negative breast adenocarcinoma have significantly improved progression-free survival with the addition of the PI3K inhibitor alpelisib to fulvestrant, which is FDA-approved.^33^ The pan-AKT inhibitor capivasertib added to fulvestrant prolonged progression-free survival vs fulvestrant alone in tumors harboring *AKT*, *PIK3CA*, or *PTEN* alterations and is expected to receive FDA approval soon. Several basket solid tumor studies of mutant selective *PIK3CA* inhibitors are underway and could be relevant to patients with MPT.^34,35^ Several tumors harbored mutations in *EGFR,* including exon 19 insertions and exon 20 insertions. These mutations are highly relevant in non-small cell lung cancer (NSCLC), epitomizing the success of precision medicine early in this tumor type. Similarly, 7% of tumors had *BRAF* V600E mutations, which are known to be targetable across several tumor types.

Perhaps the most notable finding in this series is the high rate of *TERT* promoter gene mutations, identified in 70% of samples. The telomerase reverse transcriptase (TERT) gene encodes the critical catalytic subunit of telomerase, which is a reverse transcriptase responsible for lengthening telomeres, and usually inactive in normal cells.^36-38^ In the setting of a *TERT* promoter mutation, TERT transcription is enhanced, and there is subsequent telomerase reactivation and proliferation.^37,38^ Telomerase is active primarily in progenitor and cancer cells, promoting proliferation, resisting apoptosis, and has been shown to be mutated in over 50 cancer types including melanoma, glioblastoma, NSCLC, and hepatocellular carcinoma.^21,25,38-44^

Multiple mutations in *TERT* have been described in PT. By far the most commonly identified site mutation is a −124 C > T,^18,23,25,45^ followed by −124 C > A,^45^ −146 C > T,^45^ and −75 C > T.^18^ Mutations in *TERT*, particularly in the promoter region, are associated with clinicopathologic factors. Piscuoglio et al reported that the frequency of *TERT* promoter mutations increased with increasing grade,^25^ which was confirmed by Nasir et al.^21^ Piscuoglio and others have further explored the relationship between mutational status and mRNA and protein expression, showing that *TERT* mRNA and stromal protein expression is positively correlated with *TERT* promoter hotspot mutation and/or gene amplification.^18,25^ These findings further support that the stromal component is the pathogenic driver of PT. In tumors without *TERT* promotor mutations, telomerase mRNA was undetectable in 80%, 67%, and 75% of the benign, borderline, and malignant PTs, respectively.^25^

In addition, the presence of a *TERT* mutation may be used to distinguish between fibroepithelial lesions (including both FA and PTs) with high specificity using the *TERT* promoter mutations alone, or in combination with *TERT* copy number variations.^21,23,25,45^ Nasir et al reported a sensitivity and specificity of 41% and 94% for *TERT* promoter mutations to distinguish FA from PT.^21^ In another similar study, *TERT*− 124 C > T promoter mutation and/or amplification status distinguished FA from PT with a sensitivity of 39.5% (95% CI 28.7-51.4) and a specificity of 100.0% (95% CI 95.4-100.0).^25^

While there is no direct *TERT* inhibitor, Liu et al, identified a possible therapeutic target in the FOS/GABPB/(mutant) *TERT* cascade.^42,43^ FOS is a transcriptional factor that significantly up regulates GABPB, which in turn activates the mutant *TERT* promotor. This subsequently is responsible for the mutant *TERT*-promoted oncogenesis. Liu et al, introduced a FOS inhibitor (T-5224) into cancer cell lines, inhibiting the expression of GABPB, suppressing the GABPB/mutant *TERT* cascade, reducing the control of *survivin*, activating *TRAIL-R2* (tumor necrosis factor-related apoptosis-inducing ligand receptor 2), and resulting in cancer cell apoptosis.^43^ These findings, as well as other promising studies such as targeting telomerase,^46,47^ provide hope for a potential future therapeutic strategy that warrants future clinical investigation.

While this is the largest series of genomic profiling cases to date, our series has a few notable limitations. First, central pathology review of cases at the time of sequencing was primarily to confirm the diagnosis provided by the submitting physician. Furthermore, review was done on the single provided tissue sample, which has to be presumed to be representative of the entire case with limited additional information. Although there is little concern about the phyllodes tumor diagnosis itself, there may be some cases of borderline phyllodes included in this series. As these cases were sequenced under routine clinical care, minimal information was available regarding patient-specific factors, tumor features, therapeutic interventions, or patient outcomes. Additionally, as there were no paired primary/metastatic samples in this cohort there was limited ability to describe alterations that may drive progression of disease. Lastly, the site listed as “breast” may correctly designate the tumor’s specimen location but may not reflect a lack of metastatic disease. As the primary tumor in the breast is technically more feasible for sampling, it may have been selected for sequencing but a concurrent metastatic site may exist in some cases.

## Conclusion

In the largest genomic evaluation of L/LR or metastatic MPTs to date, multiple clinically actionable mutations relevant to diverse solid tumors were found. Tumor-agnostic immunotherapy biomarkers were rare in MPTs. Routine sequencing of MPT may provide additional information to guide treatment decision-making and clinical trial enrollment. Given the rarity of MPT, multi-institutional registries and inclusion in genomically guided solid tumor basket trials may provide the best path forward to understanding targetability of genomic alterations in MPTs.

## Data Availability

The sequencing data in this study were initially generated under routine clinical care by Foundation Medicine (Cambridge, MA). The data analyzed in this study are available from Foundation Medicine; however, restrictions apply to the availability of these data, which were made available to authors and used under license for this study. The human sequence data generated in this study are not immediately publicly available due to patient privacy requirements.

## Previous presentation

Presented as an oral presentation at the Society of Surgical Oncology’s Annual Cancer Symposium on March 23, 2023.
